# Maintenance tocolysis in twin pregnancies after preterm premature rupture of membranes and neonatal outcomes: a retrospective cohort study

**DOI:** 10.1186/s12884-026-09144-8

**Published:** 2026-04-29

**Authors:** Leakana Praseth, Dan Lv, Shiyao Chen, Xufang Li, Jiaqi Han, Xinyu He, Xingguang Lin, Dongrui Deng

**Affiliations:** 1 Department of Obstetrics and Gynecology, Tongji Hospital, Tongji Medical College, Huazhong University of Science and Technology, Wuhan, Hubei 430030 China; 2 Wuxi Maternity and Child Health Care Hospital, Affiliated Women’s Hospital of Jiangnan University, Wuxi, Jiangsu China

**Keywords:** Twin pregnancies, Preterm premature rupture of membranes, Maintenance tocolysis, Perinatal outcomes, Latency, Patent ductus arteriosus

## Abstract

**Background:**

Evidence on maintenance tocolysis after preterm premature rupture of membranes (PPROM) in twin pregnancies is limited. This study evaluated the association between maintenance tocolysis and pregnancy prolongation and neonatal outcomes in this high-risk population.

**Methods:**

This retrospective cohort study included twin pregnancies with PPROM between 24 and 34 weeks at Tongji Hospital from January 2012 to December 2022, comparing maintenance tocolysis with non-maintenance tocolysis (no tocolysis or short-term tocolysis). Primary outcomes were latency and gestational age (GA) at delivery. Secondary outcomes included perinatal mortality and morbidity. Multivariate regression was fitted, and generalized estimating equations (GEE) analyses were applied to account for within-twin clustering.

**Results:**

In the main cohort, pregnancies receiving maintenance tocolysis had longer median latency (10.44 vs. 0.61 days; *p* < 0.001) despite earlier GA at PPROM. In the sub-cohort (delivery > 48 h), maintenance tocolysis remained associated with longer latency (adjusted β 5.21 days, 95% CI 1.64 − 8.79, *p* = 0.004) and higher GA at delivery (adjusted β 0.74 weeks, 95% CI 0.23 − 1.25, *p* = 0.004). Benefits varied by GA at rupture: early PPROM showed lower odds of patent ductus arteriosus (PDA: aOR 0.24, 95% CI 0.07 − 0.81, *p* = 0.021); late PPROM showed lower risk of hyperbilirubinemia (aOR 0.21, 95% CI 0.05 − 0.98, *p* = 0.047). In subgroup analyses, long-term tocolysis was superior to short-term therapy in extending pregnancy and reducing NICU admissions (aRR 0.91, 95% CI 0.85 − 0.99, *p* = 0.019) and PDA risk (aOR 0.20, 95% CI 0.06 − 0.70, *p* = 0.012). No significant increase in clinical chorioamnionitis (6.06% vs. 2.02%, *p* = 0.110) or neonatal infection was observed. However, these benefits were consistently accompanied by an elevated risk of neonatal hypoglycemia (aOR 3.75, 95% CI 1.09 − 12.95, *p* = 0.037) and fetal growth restriction in late PPROM (birthweight percentile: adjusted β -17.04%, 95% CI -30.27−-3.82, *p* = 0.012).

**Conclusions:**

In twin pregnancies with PPROM and the absence of contraindications (chorioamnionitis, placental abruption, or non‑reassuring fetal status), maintenance tocolysis effectively prolongs gestation and provides neonatal benefits that vary by rupture timing without a significant increase in infectious morbidity. These benefits must be weighed against the risks of neonatal hypoglycemia and fetal growth disturbances, underscoring a complex risk-benefit analysis for clinical decision-making.

**Supplementary Information:**

The online version contains supplementary material available at 10.1186/s12884-026-09144-8.

## Introduction

Preterm premature rupture of membranes (PPROM) refers to the rupture of fetal membranes before 37 weeks of gestation in the absence of labor. It is a major obstetric complication that contributes to nearly one-third of all preterm births and affects about 3% of pregnancies overall [1–3]. PPROM substantially increases the likelihood of adverse perinatal outcomes, primarily due to complications of prematurity, infection, placental abruption, and umbilical cord-related issues [4, 5]. The clinical challenge is further increased in twin gestations, which experience PPROM at a markedly higher rate compared to singleton pregnancies [6, 7]. Twin pregnancies complicated by PPROM are often characterized by an earlier gestational age at the time of rupture and a shorter latency period from membrane rupture to delivery, raising the risk of adverse neonatal outcomes [6–8]. Therefore, optimizing antenatal management to prolong gestation is a crucial goal in caring for these high-risk pregnancies.

Most major obstetric guidelines consistently advocate a course of antenatal corticosteroids (ACS), magnesium sulfate for neuroprotection, and prophylactic antibiotic therapy in cases of PPROM before 34 weeks’ gestation, followed by expectant management with close monitoring [4, 9–11]. This approach is supported by robust evidence demonstrating corticosteroid therapy significantly accelerates fetal organ development, substantially reducing the risks of neonatal mortality, respiratory distress syndrome (RDS), and intraventricular hemorrhage (IVH) [12–15]. Magnesium sulfate is used following PPROM once the patient is in active labor or before indicated delivery for fetal neuroprotection, particularly to reduce the rate of cerebral palsy and substantial gross motor dysfunction [16, 17]. Meanwhile, prophylactic antibiotics aim to prolong latency and lower the risk of maternal and fetal infection [18–20].

In contrast, the role of tocolytic therapy in the context of PPROM remains a subject of considerable debate. Short-term or acute tocolysis may be used to facilitate the completion of ACS administration or to enable in-utero transfer to a higher-level care facility; however, the utility of maintenance tocolysis beyond this period is highly controversial [21–23]. The primary concern is that prolonging latency may inadvertently increase the risk of intra-amniotic infection and other complications, without a demonstrable improvement in neonatal outcomes. Available research regarding maintenance tocolysis is limited and yields inconsistent conclusions, with several studies and meta-analyses in singleton pregnancies failing to show a definite advantage [21, 23–25]. This body of evidence is further limited by a lack of research specifically focusing on twin pregnancies, with many existing studies conducted before the standardization of current adjuvant therapies like ACS and antibiotics. Consequently, the reliability and external validity of evidence on the safety and efficacy of maintenance tocolysis in the contemporary management of twin pregnancies with PPROM remain limited.

In this retrospective cohort study, we sought to determine whether the use of maintenance tocolytics in twin pregnancies with PPROM before 34 weeks’ gestation is associated with a prolonged latency period and improved neonatal outcomes.

## Materials and methods

### Study design and setting

This retrospective cohort study was conducted at the Obstetrics Department of Tongji Hospital, Wuhan, China, and included twin pregnancies complicated by PPROM between 24 0/7 and 33 6/7 weeks of gestation and delivered between January 1, 2012, and December 31, 2022. The inclusion criteria were as follows: (1) establishment and maintenance of complete prenatal medical records at our hospital for the entire expectant management period following PPROM diagnosis, (2) receipt of standardized expectant management at our hospital after PPROM until delivery, and (3) a complete inpatient medical record of at least one neonate from delivery until hospital discharge or death.

The exclusion criteria comprised the following: (1) gestational age at membrane rupture less than 24 weeks or exceeding 34 weeks; (2) significant antepartum hemorrhage necessitating immediate delivery; (3) active labor (cervical dilation ≥ 4 cm) or cases requiring urgent cesarean section for a scarred uterus; (4) acute obstetric complications, including abruptio placentae and umbilical cord prolapse; (5) severe maternal pathology, such as severe preeclampsia or eclampsia, cardiopathy, severe hepatic insufficiency, or known hypersensitivity or contraindications to tocolytic agents; (6) pre-existing cervical cerclage; (7) delayed interval delivery; (8) monochorionic monoamniotic pregnancies or complications specific to monochorionic twins, including twin-to-twin transfusion syndrome (TTTS), twin reversed arterial perfusion sequence, or twin anemia-polycythemia sequence; (9) major fetal congenital anomalies or intrauterine fetal demise at the time of presentation; (10) clinical chorioamnionitis or maternal fever upon admission.

### Diagnosis of PPROM and determination of gestational age

PPROM was diagnosed either by direct observation of amniotic fluid from the cervical canal or pooling in the vaginal fornix during sterile speculum examination. When this finding was absent, diagnosis was based on vaginal fluid pH testing and ultrasound assessment for oligohydramnios. Immunoassays for soluble intercellular adhesion molecule-1 (sICAM-1) in vaginal fluid served as additional diagnostic tools [26, 27].

Gestational age (GA) was primarily determined from the last menstrual period and confirmed by ultrasonographic assessments, specifically the crown-rump length measurement in the first trimester. For patients with uncertain last menstrual period dates, second-trimester biometry was used for confirmation. In pregnancies resulting from assisted reproductive technology (ART), GA was calculated from the date of embryo transfer.

Chorionicity was determined through ultrasonography in the first or early second trimester and, when feasible, confirmed by placental pathological examination after delivery.

The latency period was defined as the time interval from the diagnosis of membrane rupture to delivery.

### Management of PPROM and tocolytic exposure

#### PPROM management

All enrolled patients received standardized expectant management following PPROM, which included ACS administration for fetal maturation, magnesium sulfate for neuroprotection, and antibiotic prophylaxis for infection prevention. The tocolytic strategy was implemented in two distinct phases: an initial acute or short-term phase, primarily designed to facilitate the completion of ACS for patients experiencing persistent uterine contractions without contraindications to tocolysis. The subsequent maintenance phase aimed to prolong pregnancy in eligible patients.

#### Tocolysis protocol and study group definitions

The protocol for the initial tocolytic agents was as follows: (1) intravenous beta-adrenergic receptor agonist (ritodrine hydrochloride) initiated at a rate of 50–100 µg/min, with dosage adjustments made until contractions were completely suppressed; (2) calcium channel blocker (nifedipine) administered orally at an initial dose of 20 mg, then 10–20 mg every 3–4 h as needed; (3) oxytocin receptor antagonist (atosiban) given as a loading dose of 6.75 mg IV over 1 min, followed by 300 µg/min for 3 h, then 100 µg/min for up to 45 h.

Maintenance tocolysis was continued in the presence of regular uterine contractions, provided there was no suspicion of chorioamnionitis, placental abruption, or non-reassuring fetal status. Upon discontinuation of the initial tocolytic, maintenance therapy, including oral ritodrine hydrochloride, atosiban, and nifedipine, was administered to regulate ongoing contractions.

Maintenance tocolysis was discontinued, and delivery was expedited upon the occurrence of any of the following: (1) development of clinical chorioamnionitis, (2) non-reassuring fetal status, (3) onset of active labor unresponsive to tocolysis, (4) maternal intolerance or adverse effects to the tocolytic agent, or (5) attainment of 34 weeks of gestation.

Based on the total duration of tocolytic administration, the cohort was divided into two groups for comparison: Maintenance tocolysis group, which received tocolytic therapy for > 48 h for the purpose of pregnancy prolongation beyond the initial acute phase, and Non-maintenance Tocolysis group, which received either no tocolysis or tocolysis for ≤ 48 h.

#### Diagnosis and monitoring of clinical chorioamnionitis

Clinical chorioamnionitis was diagnosed based on the presence of maternal fever (temperature ≥ 38℃) combined with at least two of the following criteria: purulent or foul‑smelling amniotic fluid, maternal tachycardia (> 100 beats per minute) or fetal tachycardia (> 160 beats per minute), uterine tenderness, or maternal leukocytosis (white blood cell count > 15 × 10^9^/L) [4, 28].

During maintenance tocolysis, maternal and fetal status were closely monitored daily until delivery. Vital signs (temperature, pulse) were assessed every 4–6 h, and patients were evaluated daily for uterine tenderness or abnormal vaginal discharge. Fetal heart rate was monitored twice daily. Complete blood count and C‑reactive protein levels were measured at least twice weekly, or more frequently if clinically indicated. Ultrasonographic examinations were performed every 3–4 days to assess amniotic fluid volume and fetal well-being. Non-stress tests were initiated at 32 weeks of gestation (or earlier for high-risk patients) and conducted daily. If any criterion for clinical chorioamnionitis was met, tocolysis was immediately discontinued, and delivery was expedited according to standard obstetric practice.

### Data collection

Data for this retrospective cohort study were collected through the hospital electronic medical record system. A standardized structured data extraction process was employed to collect information across four domains: (1) maternal demographics and history, (2) pregnancy course and delivery parameters, (3) details of antenatal therapeutic interventions, specifically all tocolysis therapies (including agent, timing, and duration). (4) comprehensive neonatal parameters for each offspring.

### Outcome measures

The primary outcomes were the latency period and GA at delivery. The secondary outcomes encompassed adverse perinatal outcomes, including perinatal mortality, and the following neonatal morbidities: respiratory distress syndrome (RDS), bronchopulmonary dysplasia (BPD), intraventricular hemorrhage ≥ grade 3 (IVH 3–4), necrotizing enterocolitis (NEC), retinopathy of prematurity (ROP), early-onset sepsis (EOS), late-onset sepsis (LOS), patent ductus arteriosus (PDA), pneumonia, as well as the duration of neonatal hospital stay and neonatal management measures. For the analysis of neonatal morbidities, an outcome was considered present if it occurred in at least one twin of the pair. Detailed definitions of all outcome neonatal measures are listed in Supplementary File 1 (Table S1).

### Statistical methods

#### Data handling

All statistical analyses were performed using IBM SPSS Statistics (Version 29.0; Armonk, NY, USA). Categorical variables were presented as frequencies and proportions, and were compared using Chi-Square or Fisher’s exact test, as appropriate. Normally distributed continuous variables were presented as means ± standard deviations (SD) and were analyzed using Student’s t-test. Non-normally distributed continuous variables were summarized as medians with interquartile ranges (IQR) and compared using the Mann-Whitney U test.

Data regarding twin chorionicity were missing for 36 out of 264 pregnancies (13.60%). Multiple imputation by chained equations at the maternal level was performed to generate 20 imputed datasets. During imputation, chorionicity was imputed as a pregnancy-level variable, thereby constraining both twins within a pair to share the same chorionicity status in each imputed dataset.

#### Identification of confounders

**Fig. 1:**
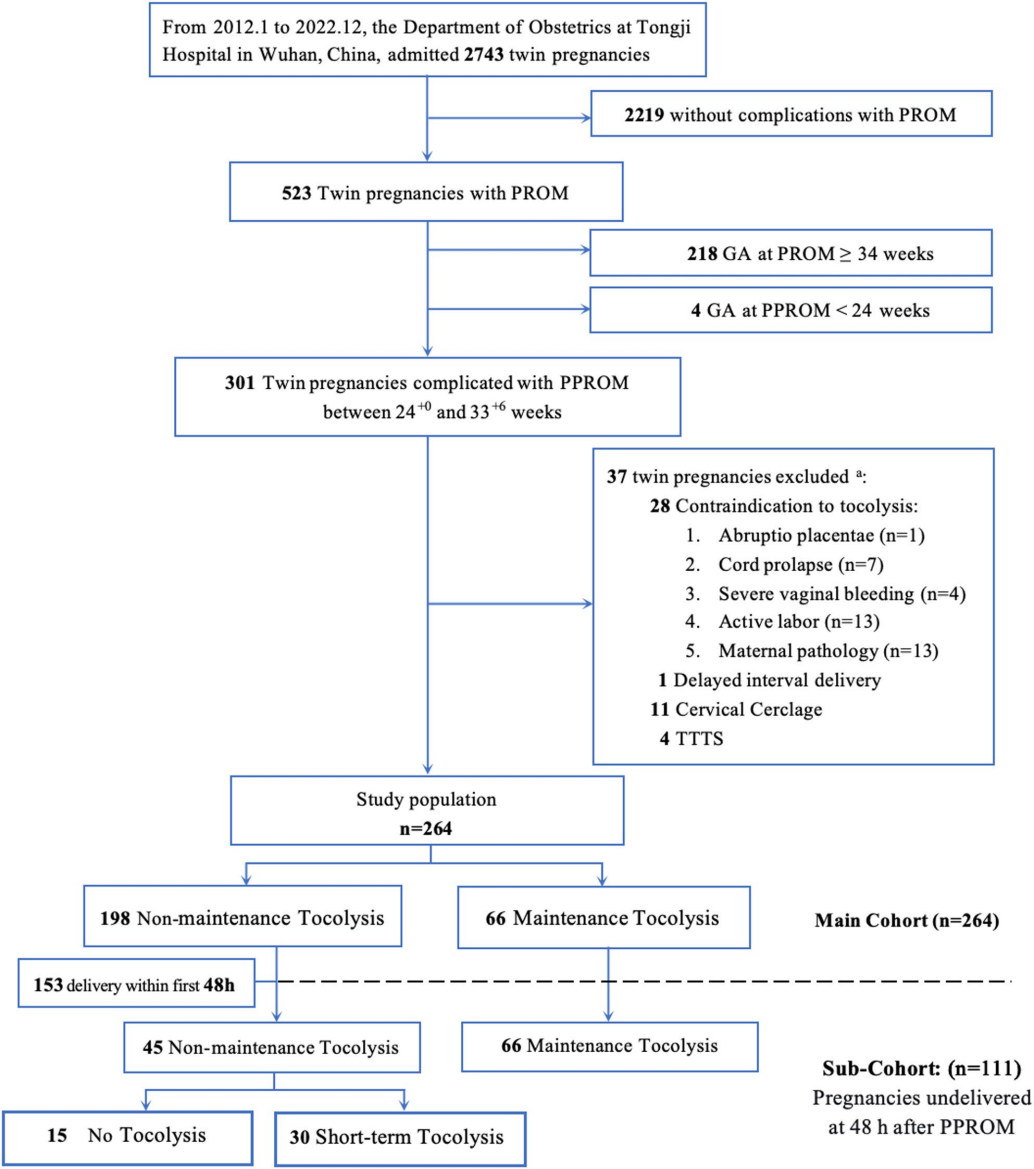
Flow chart of the study population. ^a^: Individuals could fulfill more than 1 exclusion criterion. PROM indicates premature rupture of membranes; PPROM, preterm premature rupture of membranes; GA, gestational age; TTTS, twin-to-twin transfusion syndrome

Confounders were selected as the minimally adjusted set based on the directed acyclic graph (DAG) (Supplementary File 1; Fig. 1) and a review of previous literature [6, 29]. We identified a core set of confounders, including maternal characteristics (age, method of conception, parity), chorionicity, GA at PPROM, and fetal sex.

To minimize the influence of GA on birthweight, birthweight-for-gestational-age-Z-score was calculated according to the NICHD reference for Asian neonates derived from low-risk pregnancies. The Z-score represents the number of standard deviations (SDs) a measurement deviates from the normal mean. In analyses involving birthweight, GA at delivery was consistently used for standardization and adjustment, rather than GA at PPROM, to ensure comparability. To reduce the risk of collider bias, GA at PPROM was not additionally adjusted for in models that already included birthweight measures standardized to GA at delivery.

#### Statistical analysis

Multivariate logistic regression models were employed to examine the association between maintenance tocolysis and the primary outcomes at the pregnancy level. For neonatal secondary outcomes, we used generalized estimating equations (GEE) with an exchangeable correlation structure to account for the non-independence of twin pairs within pregnancies. Binary outcomes were modeled using a binomial distribution with a logit link function. For outcomes with an observed incidence of less than 5%, Poisson regression with robust standard errors was applied to ensure reliable estimation of relative risk.

All regression analyses were performed on the 20 imputed datasets. The results were pooled according to Rubin’s rules and reported as either adjusted mean differences (β), adjusted odds ratios (aORs), or adjusted risk ratios (aRRs), each accompanied by 95% confidence intervals (CIs). A two-sided p-value < 0.05 was considered statistically significant.

### Sensitivity and subgroup analyses

To assess the robustness of our findings, we conducted a series of five sensitivity analyses, all performed within the sub-cohort of pregnancies that did not deliver within 48 h of PPROM. This restriction was applied to mitigate immortal-time bias and isolate the specific effect of maintenance tocolysis.

First, we repeated the primary analyses after excluding all pregnancies that delivered within 48 h of PPROM. The results for the main cohort and this sub-cohort are presented together in Table 4 to facilitate direct comparison. Second, recognizing that antenatal management may vary by GA at PPROM, we stratified the sub-cohort into early PPROM (24 0/7 to 31 6/7 weeks) and late PPROM (32 0/7 to 33 6/7 weeks), and conducted analyses within each subgroup. Third, to explore a potential duration-based relationship of tocolysis, we reclassified the sub-cohort into three groups based on treatment duration: no tocolysis, short-term tocolysis (≤ 48 h), and long-term tocolysis (> 48 h).

Fourth, considering the differing pharmacological profiles and clinical protocols for various tocolytic agents, we conducted an agent-specific analysis. As patients might receive more than one agent during maintenance therapy, we classified them based on the agent with the longest duration of administration to accurately reflect the predominant pharmacological exposure. Patients were assigned to Tocolytic Group 1 if a beta-adrenergic receptor agonist (ritodrine) was their longest-duration maintenance agent, and to Tocolytic Group 2 if a calcium-channel blocker (CCB, nifedipine) or an oxytocin receptor antagonist (atosiban) served that role. Finally, an exploratory analysis was performed to compare clinical outcomes between these two maintenance tocolytic agents, aiming to evaluate their comparative effectiveness.

## Results

### Study population and baseline characteristics

Between January 2012 and December 2022, 301 twin pregnancies complicated by PPROM at 24 0/7 and 33 6/7 weeks’ gestation were identified. After applying the exclusion criteria (*n* = 37), 264 twin pregnancies were included in the final analysis. Among them, 66 pregnancies (25.00%) received maintenance (long-term) tocolysis, whereas 198 (75.00%) received either no tocolysis or short-term tocolysis (Fig. 1).

Baseline maternal and obstetric characteristics are summarized in Table 1. These characteristics were comparable between groups. However, oligohydramnios was more frequent in the maintenance tocolysis group (53.03% vs. 30.30%, *p* < 0.001). The incidence of fetal distress and clinical chorioamnionitis was low and did not differ significantly between groups (12.12% vs. 15.15%, *p* = 0.544; 6.06% vs. 2.02%, *p* = 0.110, respectively). Women receiving maintenance therapy also had higher rates of prophylactic antibiotics (100.00% vs. 90.40%, *p* = 0.005) and a higher, although not statistically significant, use of magnesium sulfate (95.45% vs. 88.89%, *p* = 0.115). Antenatal corticosteroid use was high in both groups (100.00% vs. 95.96%, *p* = 0.207). No significant differences were observed in cesarean section rates, postpartum hemorrhage, or intrapartum blood loss.

**Table 1:** Comparison of clinical characteristics of twin pregnancies with PPROM stratified by maintenance tocolysis

Characteristics	Non-maintenance Tocolysis (*n* = 198)	Maintenance Tocolysis (*n* = 66)	*P*-value
Maternal characteristics			
Maternal age (years)	30.64 ± 5.07	30.05 ± 4.61	0.402
Advanced maternal age	37 (18.69)	11 (16.67)	0.712
Parity			0.299
Nulliparous	167 (84.34)	52 (78.79)	
Multiparous	31 (15.66)	14 (21.21)	
Infertility treatment			0.940
No	67 (33.84)	22 (33.33)	
Yes	131 (66.16)	44 (66.67)	
Scarred uterus	12 (6.06)	6 (9.09)	0.404
Obstetric characteristics			
Chorionicity			0.209
MCDA	24 (12.12)	3 (4.55)	
DCDA	148 (74.75)	53 (80.30)	
Missing value	26 (13.13)	10 (15.15)	
Gestational age at PPROM			**< 0.001**
24–28	12 (6.06)	15 (22.73)	
28–32	76 (38.38)	38 (57.58)	
32–34	110 (55.56)	13 (19.70)	
Gestational age at delivery			**< 0.001**
24–28	5 (2.53)	4 (6.06)	
28–32	72 (36.36)	31 (46.97)	
32–34	118 (59.6)	22 (33.33)	
34–36	3 (1.52)	9 (13.64)	
Obstetrical complications			
Anemia	15 (7.58)	8 (12.12)	0.257
GDM	52 (26.26)	17 (25.76)	0.936
ICP	10 (5.05)	2 (3.03)	0.736
HDP	18 (9.09)	1 (1.52)	0.051
Fetal distress	30 (15.15)	8 (12.12)	0.544
Oligohydramnios	60 (30.30)	35 (53.03)	**< 0.001**
Clinical chorioamnionitis	4 (2.02)	4 (6.06)	0.110
Obstetric Management			
Antibiotic			**0.005**
Yes	179 (90.4)	66 (100)	
No	19 (9.60)	0 (0.00)	
Antenatal corticosteroids			0.207
Yes	190 (95.96)	66 (100)	
No	8 (4.04)	0 (0.00)	
Magnesium sulfate			0.115
Yes	176 (88.89)	63 (95.45)	
No	22 (11.11)	3 (4.55)	
Mode of delivery			0.938
Vaginal delivery	58 (29.29)	19 (28.79)	
Cesarean delivery	140 (70.71)	47 (71.21)	
Postpartum hemorrhage	15 (7.58)	5 (7.58)	1.000
Intrapartum blood loss (*n* = 240) *	300 (200,400)	300 (200,400)	0.900

Data are expressed as mean ± standard deviation, number (%), or median (interquartile range (*IQR*))

Bold *p*‑values indicate statistical significance (*p* < 0.05)

*Abbreviations*: *PPROM* preterm premature rupture of membranes, *MCDA* monochorionic diamniotic, *DCDA* dichorionic diamniotic, *GDM* gestational diabetes mellitus, *ICP* intrahepatic cholestasis of pregnancy, *HDP* hypertensive disorders of pregnancy

*Analyzed only among patients with available data, Non-maintenance Tocolysis group (*n* = 180); Maintenance Tocolysis group (*n* = 60)

### Unadjusted primary and secondary outcomes

Women in the maintenance tocolysis group experienced PPROM at an earlier GA than those in the comparison group (29.60 ± 2.26 vs. 31.62 ± 1.88 weeks, *p* < 0.001). Despite this, the latency period was significantly longer in the maintenance tocolysis (median 10.44 days, IQR 4.95 − 16.68 vs. 0.61 days, IQR 0.28 − 1.79, *p* < 0.001). A greater proportion of pregnancies in this group remained undelivered for more than 7 days after PPROM (66.67% vs. 5.05%, *p* < 0.001). The extension of latency compensated for the earlier GA at PPROM, resulting in no significant difference in the final GA at delivery between groups (31.50 ± 2.21 vs. 31.88 ± 1.75 weeks, *p* = 0.206) (Table 2).

**Table 2:** Primary outcomes of twin pregnancies complicated by PPROM

Feature	Non-maintenance Tocolysis (*n* = 198)	Maintenance Tocolysis (*n* = 66)	*P*-value
GA at PPROM (weeks)	31.62 ± 1.88	29.60 ± 2.26	**< 0.001**
GA at delivery (weeks)	31.88 ± 1.75	31.50 ± 2.21	0.206
Prolongation of pregnancy			
Latency duration (days)	0.61 (0.28,1.79)	10.44 (4.95,16.68)	**< 0.001**
Latency period			**< 0.001**
Up to 72 h	163 (82.32)	4 (6.06)	
3–7days	25 (12.63)	18 (27.27)	
Over 7days	10 (5.05)	44 (66.67)	

Data are expressed as mean ± standard deviation, median (interquartile range (*IQR*)), or number (%)

Bold *p*‑values indicate statistical significance (*p* < 0.05)

*Abbreviations*: *GA* gestational age, *PPROM* preterm premature rupture of membranes

Neonatal outcomes were evaluated in 474 neonates (353 in the non-maintenance group and 121 in the maintenance group), after excluding intrapartum stillbirth (*n* = 2), declined neonatal resuscitation (*n* = 6), and those with incomplete NICU medical records (*n* = 46). Compared to the non-maintenance group, maintenance tocolysis was associated with a lower rate of NICU admission (91.74% vs. 98.30%, *p* = 0.002) and a lower incidence of neonatal hyperbilirubinemia (64.64% vs. 73.94%, *p* = 0.046) (Table 3).

**Table 3:** Secondary outcomes of twin pregnancies with PPROM

Feature	Non-maintenance Tocolysis	Maintenance Tocolysis	*P*-value
Neonatal characteristics ^a^			
5-min Apar score < 7 (*n* = 528)	44 (11.11)	21 (15.91)	0.146
Birthweight (g) (*n* = 497)	1696 ± 366	1588 ± 392	**0.006**
Birthweight percentile	28.10 (9.68,49.43)	21.60 (6.65,41.70)	**0.041**
Birthweight Z-score	-0.57 (-1.28, -0.01)	-0.78 (-1.50, -0.21)	**0.036**
Birthweight < 1,000 g (*n* = 497)	16 (4.26)	5 (4.13)	0.953
Neonatal genders (*n* = 264)			0.947
Male-male	82 (41.41)	27 (40.91)	
Female-female	48 (24.24)	15 (22.73)	
Male-female	68 (34.34)	24 (36.36)	
Neonatal management ^b^ (*n* = 474)			
NICU admission rate	347 (98.30)	111 (91.74)	**0.002**
Neonatal hospital stay (days)	18.00 (11.50,32.00)	24.00 (11.00,37.50)	0.357
Mechanical ventilation	94 (26.63)	36 (29.75)	0.506
CPAP use	185 (52.41)	62 (51.24)	0.824
Surfactant use	92 (26.06)	34 (28.1)	0.662
Neonatal complications ^b^ (*n* = 474)			
Hyperbilirubinemia	261 (73.94)	78 (64.46)	**0.046**
Pneumonia	143 (40.51)	55 (45.45)	0.341
RDS	137 (38.81)	52 (42.98)	0.419
Anaemia	97 (27.48)	34 (28.1)	0.895
PDA	49 (13.88)	9 (7.44)	0.062
BPD	46 (13.03)	23 (19.01)	0.108
Hypoglycemia	23 (6.52)	18 (14.88)	**0.005**
IVH (3–4)	20 (5.67)	8 (6.61)	0.703
ROP	12 (3.40)	1 (0.83)	0.199
NEC	8 (2.27)	5 (4.13)	0.332
Early-onset Sepsis	3 (0.85)	4 (3.31)	0.074
Late-onset Sepsis	14 (3.97)	3 (2.48)	0.579
Neonatal death	29 (8.22)	10 (8.26)	0.986

Data are expressed as mean ± standard deviation, number (%), or median (interquartile range (*IQR*)). The neonatal outcome was considered present if it occurred in at least one neonate of the twin pair

Bold *p*‑values indicate statistical significance (*p* < 0.05)

*Abbreviations*: *NICU* neonatal intensive care unit, *CPAP* continuous positive airway pressure, *RDS* respiratory distress syndrome, *BPD* bronchopulmonary dysplasia, *PDA* patent ductus arteriosus, *IVH* intraventricular hemorrhage, *NEC* necrotizing enterocolitis, *ROP* retinopathy of prematurity

^a^ Analyzed for both twins with available data

^b^ Neonatal complications excluded intrapartum stillbirth (*n* = 2), declined neonatal resuscitation (*n* = 6), and lost follow-up or incomplete information from the full NICU records (*n* = 46)

Conversely, neonates in the maintenance group had a higher incidence of hypoglycemia (14.88% vs. 6.52%, *p* = 0.005) and lower mean birthweight (1588 ± 392 g vs. 1696 ± 366 g, *p* = 0.006), with lower median birthweight Z-score (-0.78 SD, IQR − 1.50−-0.21 vs. -0.57 SD, IQR − 1.28−-0.01, *p* = 0.036) and lower birthweight percentiles (21.60%, IQR 6.65 − 41.70 vs. 28.10%, IQR 9.68 − 49.43, *p* = 0.041). No significant differences were observed in duration of neonatal hospitalization or the need for respiratory support, including mechanical ventilation, nasal continuous positive airway pressure (nCPAP), or surfactant administration (Table 3).

### Adjusted analysis

#### Main cohort analysis

After adjusting for confounders, maintenance tocolysis was independently associated with a significantly longer latency period (adjusted β 8.84 days, 95% CI 6.90 − 10.78, *p* < 0.001) and a higher GA at delivery (adjusted β 1.27 weeks, 95% CI 0.99 − 1.54, *p* < 0.001). This prolongation corresponded with lower risk of several neonatal outcomes, including NICU admission (aRR 0.90, 95% CI 0.83 − 0.98, *p* = 0.016), neonatal death (aOR 0.35, 95% CI 0.13 − 0.92, *p* = 0.033), RDS (aOR 0.45, 95% CI 0.23 − 0.88, *p* = 0.021), PDA (aOR 0.22, 95% CI 0.09 − 0.54, *p* = 0.001), and ROP prematurity (aOR 0.07, 95% CI 0.01 − 0.82, *p* = 0.034) (Table 4).

**Table 4:** Association between maintenance tocolysis after PPROM and outcomes in the main cohort and sub-cohort

Outcome	Main Cohort (*n* = 264)		Sub-Cohort (*n* = 111)	
	Maintenance Tocolysis vs. Non-maintenance Tocolysis (ref)		Maintenance Tocolysis vs. Non-maintenance Tocolysis (ref)	
	Adjusted β (95% CI)	*P-value*	Adjusted β (95% CI)	*P-value*
GA at delivery ^a^	1.27 (0.99,1.54)	**< 0.001**	0.74 (0.23,1.25)	**0.004**
Latency prolongation ^a^	8.84 (6.90,10.78)	**< 0.001**	5.21 (1.64,8.79)	**0.004**
Birthweight ^b^	-52.84 (-102.86, -2.82)	**0.038**	-11.75 (-73.27,49.77)	0.708
Birthweight percentile ^c^	-5.72 (-11.48,0.05)	0.052	-2.16 (-9.90,5.57)	0.584
Birthweight Z-score ^c^	-0.25 (-0.51,0.01)	0.059	-0.07 (-0.41,0.28)	0.709
Neonatal hospital stay ^a^	-0.14 (-0.30,0.03)	0.099	-0.07 (-0.29,0.15)	0.544
Latency period ^a^	aOR (95% CI)	*P*	aOR (95% CI)	*P*
Up to 72 h	reference		reference	
3–7days	26.73 (8.10,88.23)	**< 0.001**	1.92 (0.49,7.62)	0.352
Over 7days	139.39 (37.76,514.64)	**< 0.001**	10.41 (2.41,44.90)	**0.002**
3–7days	reference		reference	
Over 7days	5.21 (1.90,14.30)	**0.001**	5.41 (1.96,14.91)	**0.001**
Neonatal complications ^a^	aRR/aOR (95% CI)	*P*	aRR/aOR (95% CI)	*P*
NICU admission	0.90 (0.83,0.98)	**0.016**	0.92 (0.85,1.01)	0.074
Hyperbilirubinemia	0.82 (0.47,1.44)	0.482	0.91 (0.41,2.02)	0.823
Pneumonia	0.93 (0.52,1.65)	0.804	1.06 (0.51,2.22)	0.870
RDS	0.45 (0.23,0.88)	**0.021**	0.72 (0.31,1.69)	0.453
Anaemia	0.60 (0.31,1.16)	0.128	0.64 (0.30,1.37)	0.252
PDA	0.22 (0.09,0.54)	**0.001**	0.20 (0.06,0.68)	**0.010**
BPD	0.56 (0.22,1.4)	0.216	0.63 (0.23,1.73)	0.373
Hypoglycemia	2.65 (1.14,6.18)	**0.024**	3.75 (1.09,12.95)	**0.037**
IVH (3–4)	0.68 (0.23,2.02)	0.493	0.46 (0.15,1.45)	0.187
ROP	0.07 (0.01,0.82)	**0.034**	0.02 (0.00,2.67)	0.113
NEC	0.79 (0.20,3.15)	0.743	0.64 (0.14,2.90)	0.560
Early-onset Sepsis	1.44 (0.20,10.57)	0.719	0.65 (0.11,3.98)	0.644
Late-onset Sepsis	0.29 (0.06,1.44)	0.130	0.38 (0.07,2.03)	0.257
Neonatal death	0.35 (0.13,0.92)	**0.033**	0.32 (0.08,1.34)	0.119

Bold p‑values indicate statistical significance (p < 0.05)

The neonatal outcome was considered present if it occurred in at least one neonate of the twin pair

Main cohort *n* = 264 pregnancies (Non-maintenance: *n* = 198; Maintenance: *n* = 66); 474 neonates (Non-maintenance: *n* = 353; Maintenance: *n* = 121); Sub-cohort *n* = 111 pregnancies (Non-maintenance: *n* = 45; Maintenance: *n* = 66); 198 neonates (Non-maintenance: *n* = 77; Maintenance: *n* = 121)

*CI* confidence interval, *aRR* adjusted relative risk, *aOR* adjusted odds ratio, *ref* reference group

^a^ Adjusted for maternal characteristics (age, method of conception, parity), chorionicity, GA at PPROM, and tocolytic therapy

^b^ Adjusted for maternal characteristics (age, method of conception, parity), chorionicity, GA at delivery, tocolytic therapy, and fetal sex

^c^ Adjusted for maternal characteristics (age, method of conception, parity), chorionicity, tocolytic therapy, and fetal sex

However, maintenance tocolysis was also associated with lower absolute birthweight (adjusted β -52.84 g, 95%CI -102.86 to -2.82, *p* = 0.038), though the associations with birthweight percentile (adjusted β -5.72%, 95% CI -11.48 − 0.05, *p* = 0.052) and birthweight Z-score (adjusted β -0.25 SD, 95% CI -0.51 − 0.01, *p* = 0.059) were not statistically significant after adjustment. Furthermore, we observed the increasing odds of neonatal hypoglycemia (aOR 2.65, 95% CI 1.14 − 6.18, *p* = 0.024) in the maintenance group. The initial observed association with neonatal hyperbilirubinemia was no longer significant (aOR 0.82, 95% CI 0.47 − 1.44, *p* = 0.482). No significant associations with EOS, LOS, or neonatal pneumonia were observed after adjustment (Table 4).

#### Sub-cohort excluding deliveries ≤ 48 h

To strengthen the robustness of our findings, we performed a sensitivity analysis on a sub-cohort of 111 pregnancies that did not deliver within 48 h after PPROM diagnosis. This sub-cohort included 66 pregnancies (121 neonates) in the maintenance tocolysis group and 45 (77 neonates) in the non-maintenance group (Fig. 1). In this sub-cohort, maintenance tocolysis remained associated with prolonged latency (adjusted β 5.21 days, 95% CI 1.64 − 8.79, *p* = 0.004) and higher GA at delivery (adjusted β 0.74 weeks, 95% CI 0.23 − 1.25, *p* = 0.004). It also confirmed a significant reduction in PDA incidence (aOR 0.20, 95% CI 0.06 − 0.68, *p* = 0.010).

Notably, the association with reduced neonatal birthweight was attenuated and no longer significant (adjusted β -11.75 g, 95% CI -73.27 − 49.77, *p* = 0.708). However, the increased risk of neonatal hypoglycemia persisted (aOR 3.75, 95% CI 1.09 − 12.95, *p* = 0.037), while reductions in other neonatal complications, such as RDS, ROP, and neonatal death, were not observed. No association of neonatal infection outcomes, including EOS (aOR 0.65, 95% CI 0.11 − 3.98, *p* = 0.664), LOS (aOR 0.38, 95% CI 0.07 − 2.03, *p* = 0.257), and neonatal pneumonia (aOR 1.06, 95% CI 0.51 − 2.22, *p* = 0.870), was observed (Table 4).

#### Subgroup analysis by GA at PPROM

Within the sub-cohort, pregnancies were stratified into early PPROM (24 0/7 to 31 6/7 weeks; *n* = 81 pregnancies, 144 neonates) and late PPROM (32 0/7 to 33 6/7 weeks; *n* = 30 pregnancies, 54 neonates). Maintenance tocolysis was administered more frequently in the early PPROM subgroup (65.43%, 53/81) than in the late PPROM subgroup (43.33%, 13/30). The results after adjustment consistently confirmed the association between maintenance tocolysis and a significantly longer latency period (early: adjusted β 6.01 days, 95% CI 1.18 − 10.85, *p* = 0.015; late: adjusted β 5.34 days, 95% CI 2.44 − 8.25, *p* < 0.001) and a higher GA at delivery (early: adjusted β 0.85 weeks, 95% CI 0.16 − 1.54, *p* = 0.016; late: adjusted β 0.77 weeks, 95% CI 0.35 − 1.20, *p* < 0.001) across both strata (Supplementary File 2: Table S1).

Additionally, in the early PPROM subgroup, maintenance tocolysis was specifically associated with a lower risk of PDA (aOR 0.24, 95% CI 0.07 − 0.81, *p* = 0.021). In the late PPROM subgroup, it was associated with reduced risks of neonatal hyperbilirubinemia (aOR 0.21, 95% CI 0.05 − 0.98, *p* = 0.047) and severe IVH (aOR 0.07, 95%CI 0.005 − 0.90, *p* = 0.042).

However, in the late PPROM subgroup, maintenance therapy was also associated with a lower neonatal birthweight percentile (adjusted β -17.04%, 95% CI -30.27−-3.82, *p* = 0.012) along with non-significant neonatal absolute birthweight (adjusted β -74.63 g, 95% CI -183.33 − 34.07, *p* = 0.178) and birthweight Z-score (adjusted β -0.38 SD, 95% CI -0.88 − 0.12, *p* = 0.138) (Supplementary File 2: Table S1).

#### Duration-based analysis

A duration-based analysis was conducted in the sub-cohort. Long-term therapy (*n* = 66 pregnancies, 121 neonates) demonstrated superior benefits compared to short-term therapy (*n* = 30 pregnancies, 53 neonates), with significant associations with prolonged latency (adjusted β 5.61 days, 95% CI 1.59 − 9.64, *p* = 0.006), higher GA at delivery (adjusted β 0.80 weeks, 95% CI 0.22 − 1.37, *p* = 0.007), reduced NICU admission risk (aRR 0.91, 95% CI 0.85 − 0.99, *p* = 0.019) and lower PDA risk (aOR 0.20, 95% CI 0.06 − 0.70, *p* = 0.012) (Supplementary File 2: Table S2).

When compared directly to no tocolytic (*n* = 15 pregnancies, 24 neonates), long-term therapy was associated with a lower risk of neonatal ROP (aRR 0.003, 95% CI 0.000 − 0.46, *p* = 0.023), although pregnancy prolongation did not significantly differ. No significant differences were observed between the short-term tocolysis and no tocolysis (Supplementary File 2: Table S2).

#### Agent-specific analysis

When stratified by tocolytic agent with the longest duration of use, maintenance tocolysis was associated with favorable neonatal outcomes, though the magnitude of associations varied by drug class. For both agent classes, maintenance therapy was associated with longer latency and higher GA at delivery; however, these associations did not reach statistical significance. Specifically, adjusted β estimates for latency were 5.30 days (95% CI -0.14 − 10.75, *p* = 0.056) in Group 1 (beta-adrenergic agonist, ritodrine; *n* = 65 pregnancies, 120 neonates) and 6.80 days (95% CI -3.16 − 16.75, *p* = 0.181) in Group 2 (CCB, nifedipine or oxytocin receptor antagonist, atosiban; *n* = 30 pregnancies, 52 neonates). Corresponding adjusted β estimates for GA at delivery were 0.76 weeks (95% CI -0.02 − 1.53, *p* = 0.057) and 0.97 weeks (95% CI -0.45 − 2.39, *p* = 0.182), respectively (Supplementary File 2: Table S3).

Beta-agonist-based maintenance therapy was associated with a higher likelihood of delivery beyond 7 days (aOR 16.88, 95% CI 2.36 − 120.63, *p* = 0.005) and a lower risk of NICU admissions (aRR 0.88, 95% CI 0.79 − 0.99, *p* = 0.030). CCB or oxytocin antagonist-based maintenance therapy was associated with lower odds of severe IVH (aOR 0.003, 95% CI 0.000 − 0.58, *p* = 0.031) and neonatal death (aOR 0.01, 95% CI 0.001 − 0.18, *p* = 0.002) (Supplementary File 2: Table S3).

In direct comparison, the CCB/oxytocin antagonist group (*n* = 20 pregnancies, 34 neonates) showed a lower rate of neonatal pneumonia (aOR 0.10, 95%CI 0.02 − 0.51, *p* = 0.006), but a substantially higher risk of late-onset sepsis (aOR 24.75, 95%CI 5.09 − 120.48, *p* < 0.001) compared to the beta-agonist group (*n* = 46 pregnancies, 87 neonates) (Supplementary File 2: Table S4).

## Discussion

### Principal finding and context

This study demonstrates that in twin pregnancies complicated by PPROM between 24 0/7 and 33 6/7 weeks of gestation, maintenance tocolysis presents a dualistic clinical profile. In the main cohort, maintenance tocolysis was associated with significantly longer latency and higher GA at delivery after adjustment. The effect largely compensated for the earlier GA at PPROM observed in the maintenance group.

This successful temporization translated into several neonatal benefits, including reduced rates of NICU admission and specific neonatal morbidities such as PDA in early PPROM and hyperbilirubinemia in the late PPROM subgroup. Importantly, these benefits were observed without a significant increase in clinical chorioamnionitis or neonatal sepsis, supporting the safety of the protocol when strict selection criteria are applied. However, these advantages were accompanied by a higher incidence of neonatal hypoglycemia and evidence of reduced birthweight percentiles in late PPROM. Findings from subgroup and sensitivity analyses suggest that both benefits and risks vary according to gestational age at rupture, duration of therapy, and the specific agent used.

### Interpretation

#### Pregnancy prolongation

Our analysis provides compelling evidence that maintenance tocolysis effectively achieves its fundamental efficacy of prolonging pregnancy following PPROM in twin gestations. The magnitude of this effect was substantial, extending the median latency period from less than one day in the controls to over ten days in the treatment group, an absolute difference that remained highly significant after multivariate adjustment. This prolongation was sufficient to compensate for the earlier GA at PPROM in the treatment group, resulting in a higher final delivery GA after adjustment. These findings align with the biological goal of tocolytic therapy and are consistent with evidence from single pregnancies [21].

Further sensitivity analysis refined this understanding. Within the sub-cohort of 111 pregnancies that did not deliver within the first 48 h, a group already past the highest-risk period for immediate delivery [10, 30], maintenance tocolysis remained associated with a significant, though attenuated, prolongation of over five days and higher GA at delivery. This confirms an ongoing therapeutic effect beyond acute stabilization. Furthermore, the duration-based analysis, showing greater prolongation with long-term versus short-term therapy, supports the concept that sustained suppression of uterine activity is key to maximizing latency.

#### Prevention of early delivery and promotion of fetal maturation

The significant reductions in critical neonatal outcomes observed in the main cohort, including NICU admission, neonatal death, RDS, PDA, and ROP, are best interpreted as the direct outcome of preventing delivery at the limits of viability [30, 31], shifting the GA spectrum upward and thereby attenuating the severe complications tied to extreme immaturity [10, 32].

For pregnancies that achieve initial stabilization, the benefits become more outcome-specific and maturation-dependent. PDA is more common at earlier gestational age [33]. The consistent reduction in PDA incidence, observed even after excluding immediate deliveries and particularly in the long-term therapy within the early PPROM subgroup, is a noteworthy finding. As a marker of cardiovascular immaturity, PDA reduction may reflect the synergistic benefits of pregnancy prolongation and adequate exposure to ACS. The physiological closure of the ductus arteriosus (DA) after birth is promoted by increased oxygen and decreased prostaglandin levels [33]. Extended latency may ensure sufficient time for ACS to exert its full effect. Experimental and clinical evidence suggest that ACS promotes DA closure by enhancing its contractile sensitivity to oxygen while simultaneously reducing prostaglandin sensitivity and synthesis [34–36]. Therefore, the reduction in PDA serves as a specific indicator of successful biochemical preparation for extrauterine life.

Additional context-specific benefits emerged in subgroup analyses. In the late PPROM subgroup, maintenance tocolysis was associated with reduced risks of severe IVH (grade 3–4) and hyperbilirubinemia. The reduction in severe IVH (aOR 0.07, 95% CI 0.005 − 0.90, *p* = 0.042) is based on a small subgroup (*n* = 30 pregnancies, 54 neonates) and carries a wide confidence interval, warranting cautious interpretation. In agent‑specific analyses, a similar pattern was observed for CCB or oxytocin antagonist‑based maintenance therapy (aOR 0.003, 95% CI 0.000 − 0.58, *p* = 0.031), though this estimate derives from an even smaller subgroup (*n* = 30 pregnancies, 52 neonates) and is statistically unstable. Consequently, neither finding should be interpreted as definitive evidence of a protective effect.

Nevertheless, these exploratory findings align with previous studies suggesting plausible mechanisms. The lower risk of severe IVH may be explained by securing additional time for neuroprotective agents such as magnesium sulfate [17, 37] or ACS [38], or by beneficial effects on fetal cerebral hemodynamics or direct anti-inflammatory properties associated with specific tocolytic agents like CCB or oxytocin receptor antagonists, which could mitigate secondary brain injury in the inflammatory context of PPROM [39, 40]. Although our study was not powered to definitively assess these agent‑specific effects, these mechanistic insights provide a biological framework that warrants further investigation in larger, adequately powered studies.

Regarding reduced hyperbilirubinemia in late PPROM, it likely indicates enhanced fetal hepatic maturation due to extended gestation. The activity of the critical bilirubin-conjugating enzyme is profoundly low in preterm infants, with only minimal activity present at 32 weeks’ gestation [41]. Each additional day of latency beyond 32 weeks incrementally enhances the fetal liver’s capacity to metabolize bilirubin, thereby translating into a lower risk of clinical hyperbilirubinemia at birth.

#### Duration of tocolysis

The duration-based analysis provided critical insights for therapeutic optimization. Long-term maintenance tocolysis demonstrated clear superiority over short-term therapy in prolonging latency, increasing GA at delivery, and reducing both NICU admission and PDA rates. This pattern supports the concept that sustained suppression of uterine activity may be necessary to achieve the incremental fetal maturation required to meaningfully reduce the need for intensive care [31]. However, confounding by indication cannot be excluded. Women who received long-term tocolysis were those who responded well to initial acute tocolysis and may have had a less aggressive or later-presenting form of uterine contraction or amniotic fluid leak.

In an exploratory comparison between long‑term tocolysis and no tocolysis, a lower risk of ROP was observed (aRR 0.003, 95% CI 0.000 − 0.46, *p* = 0.023). This estimate is highly unstable due to the sparse number of ROP events in this subgroup. However, previous studies have demonstrated the benefits of ACS at the very earliest, highest-risk gestational ages for retinal vascular development [42, 43]. Each extra day in utero allows for further physiological development of the retinal vasculature [44], reducing the susceptibility to the dysregulated postnatal angiogenesis that characterizes ROP.

#### Agent-related differences

The agent-specific analyses revealed divergent profiles unrelated to tocolytic effects that warrant consideration. Beta-agonist (ritodrine) therapy was strongly correlated with achieving over 7 days of prolonged latency, underscoring its efficacy as a tocolytic agent and its role in reducing the NICU admission rate.

As noted in the subgroup analysis (Sect.  4.2.2), CCB or oxytocin receptor antagonist-based maintenance therapy was associated with lower odds of severe IVH in exploratory analyses. Additionally, this agent group was associated with lower odds of neonatal mortality (aOR 0.01, 95% CI 0.001 − 0.18, *p* = 0.002). Both estimates derive from a small subgroup (Group 2, *n* = 30 pregnancies, 52 neonates) and are accompanied by wide confidence intervals. Therefore, they should be considered as exploratory and hypothesis‑generating rather than definitive evidence of effect.

Moreover, a divergent neonatal infection profile emerged. Compared to the beta-agonist (ritodrine), the CCB/oxytocin antagonist regimen was linked to lower rates of neonatal pneumonia, potentially due to enhanced lung maturation and anti-inflammatory modulation of the fetal lung environment [45, 46]. Conversely, the increased late-onset sepsis observed with this regimen is likely a hazard inherent to significantly prolonging gestation in the context of PPROM; again, the wide confidence interval (95% CI 5.09 − 120.48) underscores the imprecision of this estimate. These observations indicate that the risk-benefit profile may be pharmacologically tailored; however, they also necessitate rigorous sepsis surveillance when using these agents. Given the limited sample size in these subgroup analyses, all agent‑specific findings should be interpreted as exploratory and require validation in larger studies.

#### Metabolic and growth concerns

Counterbalancing the neonatal benefits are two notable safety signals: neonatal hypoglycemia and effects on fetal growth. The increased risk of neonatal hypoglycemia remained robust in the sub-cohort sensitivity analyses, confirming it as a direct consequence of the therapeutic regimen. The observed risk aligns with the established metabolic effects of ACS [47], which were administered to nearly all patients in our cohort (100% in the maintenance group vs. 95.96% in controls). As demonstrated in a large study of twin pregnancies [48], ACS therapy is independently associated with increased risks of neonatal hypoglycemia. This suggests that in our population, the nearly universal ACS coverage likely contributed to the metabolic disturbance.

Additionally, a direct pharmacological mechanism is implicated for beta-adrenergic agonists like ritodrine, which stimulate maternal pancreatic receptors, thereby increasing insulin secretion and inducing maternal hyperglycemia. Consequently, elevated glucose levels traverse the placenta, leading to chronic fetal hyperglycemia [49]. As a result, the fetal pancreas undergoes compensatory hyperinsulinemia, placing the neonate in a state of relative hyperinsulinemia at birth. The abrupt cessation of maternal glucose supply subsequently precipitates hypoglycemia [50].

The non-significant agent-specific result for ritodrine (aOR 2.17, 95% CI 0.46 − 10.18, *p* = 0.324) is likely attributable to reduced statistical power (Group 1, *n* = 65 pregnancies, 120 neonates) upon stratification rather than a refutation of this well-established pathway [51], given that the consistent signal observed in aggregated analyses robustly supports a class-level effect. Therefore, neonatal hypoglycemia represents an inherent metabolic liability associated with maintenance tocolysis, particularly with beta-agonists. This underscores the importance of vigilant monitoring of neonatal glucose levels in all affected infants and emphasizes that such therapy should be reserved for cases, such as early PPROM, where the benefits of prolongation clearly outweigh the associated risks.

The observed association between maintenance tocolysis with a lower birthweight percentile in late PPROM suggests a specific effect on fetal growth proportionality. This finding is a coherent consequence of the therapeutic strategy, wherein pregnancy prolongation increases fetal exposure to ACS, which has a well-established, dose-dependent growth-restrictive effect [38, 52]. The stronger association in late PPROM, compared to early PPROM, is mechanistically explained by differential ACS exposure. Pregnancies with late PPROM are more likely to receive extended or 2nd courses of ACS [47], whereas management in early PPROM is often focused on a single course with imminent delivery anticipated. Therefore, the significantly greater cumulative fetal exposure to ACS in late PPROM primarily explains the more pronounced shift in the growth trajectory.

Moreover, this effect is compounded by the pro-inflammatory intrauterine environment of prolonged PPROM [53]. Biomarkers such as PARK-7 (DJ-1) are elevated in PPROM and correlate directly with impaired fetal growth [54], indicating that subclinical inflammation constrains placental function and fetal nutrition [55]. Thus, maintenance tocolysis creates a dual burden: the intended growth-restrictive effect of ACS is superimposed upon a pre-existing, inflammation-driven constraint. This represents a calculated clinical trade-off, wherein a measurable impact on fetal growth is accepted to secure the substantial benefits of advanced gestational age. These findings underscore the necessity for serial growth surveillance in all twin pregnancies undergoing prolonged management for PPROM.

#### Absolute infection increase

Although the incidence of clinical chorioamnionitis did not differ significantly between the maintenance and non‑maintenance groups (6.06% vs. 2.02%, *p* = 0.110), the approximately 4% absolute increase in the maintenance group warrants careful consideration. This numerical difference may reflect a modest but genuine increase in infection risk associated with prolonged latency after PPROM [21], or it may be attributable to chance, given the limited sample size and low overall event rate. Importantly, all cases of chorioamnionitis were identified early through the daily monitoring protocol described in the Methods, and tocolysis was promptly discontinued upon diagnosis. No maternal deaths or severe septic complications occurred in either group. Therefore, the decision to use maintenance tocolysis should involve a careful risk‑benefit assessment: the substantial benefits of pregnancy prolongation must be weighed against a potential increase in infectious morbidity. In clinical practice, this risk can be minimized by adhering to strict selection criteria and maintaining vigilant daily monitoring for early signs of infection.

### Strengths and limitations

This study includes a substantial cohort of twin pregnancies with PPROM managed under contemporary obstetric protocols, including ACS, antibiotic prophylaxis, and magnesium sulfate, ensuring that our findings reflect current clinical practice. The robustness of our results is supported by multivariate regression with careful adjustment for potential confounders, further strengthened by GEE to account for the correlated outcomes within twin pairs. This methodology enabled a more accurate assessment of the independent effect of maintenance tocolysis. Furthermore, comprehensive sensitivity analyses were performed to address clinical heterogeneity, specifically variations in treatment duration, GA at PPROM, and specific pharmacological agents, thereby reinforcing the validity of our conclusions and providing a nuanced perspective on the therapy’s effects across diverse clinical scenarios.

Several limitations should be considered. First, the retrospective single-center design limits causal inference and may introduce residual confounding and center-specific practice variations. Second, while the main cohort (264 pregnancies, 474 neonates) provided sufficient power for primary analyses, the sample sizes in subgroup analyses, particularly for late PPROM (*n* = 30 pregnancies, 54 neonates) and agent‑specific comparisons (Group 2, *n* = 30 pregnancies, 52 neonates), are modest. Consequently, estimates for rare outcomes (e.g., ROP, severe IVH) in these subgroups have very wide confidence intervals and are statistically unstable; these stratified results should be considered exploratory and hypothesis‑generating. Third, the inclusion of both local and transferred patients may introduce selection bias. Fourth, we were unable to analyze outcomes separately by chorionicity due to insufficient sample size. Finally, the lack of long‑term neurodevelopmental outcome data remains an important gap for future investigation.

## Conclusion

In twin pregnancies complicated by PPROM, maintenance tocolysis effectively prolongs gestation and reduces several prematurity‑related complications without increasing clinical chorioamnionitis when applied under strict selection criteria (absence of chorioamnionitis, placental abruption, non‑reassuring fetal status, and active labor). This intervention exerts a consistent effect in extending the latency period, most critically preventing immediate delivery within the first 48 h; thereby attenuating the incidence of major prematurity-related complications. However, these benefits are counterbalanced by an increased risk of neonatal hypoglycemia and potential growth effects, particularly in late PPROM. Therefore, clinical implementation should be selective, coupled with vigilant monitoring. The nuanced risk‑benefit profile underscores the need for individualized treatment strategies and highlights the necessity for future large-scale studies to define optimal patient selection and tocolytic regimens.

## Supplementary Information

Supplementary Material 1.

Supplementary Material 2.

## Data Availability

The datasets used in the present study are available from the corresponding author upon reasonable request.

## Abbreviations

ACS: Antenatal corticosteroids
ART: Assisted reproductive technology
BPD: Bronchopulmonary dysplasia
CCB: Calcium channel blocker
DCDA: Dichorionic diamniotic
GA: Gestational age
GEE: Generalized estimating equations
IVH: Intraventricular hemorrhage
MCDA: Monochorionic diamniotic
nCPAP: Nasal continuous positive airway pressure
NEC: Necrotizing enterocolitis
NICU: Neonatal intensive care unit
PDA: Patent ductus arteriosus
PPROM: Preterm premature rupture of membranes
RDS: Respiratory distress syndrome
ROP: Retinopathy of prematurity
TTTS: Twin-to-twin transfusion syndrome
