# Supplementary material for: Maintenance tocolysis in twin pregnancies after preterm premature rupture of membranes and neonatal outcomes: a retrospective cohort study
Source: BMC Pregnancy Childbirth. 2026 Apr 29;26:647. doi: 10.1186/s12884-026-09144-8 (PMC13267740; doi:10.1186/s12884-026-09144-8)
Supplement: Supplementary file 1 — Supplementary Material 1. [file 12884_2026_9144_MOESM1_ESM.docx]

**Maintenance tocolysis in twin pregnancies after preterm premature rupture of membranes and neonatal outcomes: a retrospective cohort study**

Leakana Praseth^a^, Dan Lv ^a,b^, Shiyao Chen^a^, Xufang Li^a^, Jiaqi Han^a^, Xinyu He^a^, Xingguang Lin^a,^ ^†^, Dongrui Deng ^a,^ ^†, *^

a Department of Obstetrics and Gynecology, Tongji Hospital, Tongji Medical College, Huazhong University of Science and Technology, 430030, Wuhan, Hubei, China

b Wuxi Maternity and Child Health Care Hospital, Affiliated Women’s Hospital of Jiangnan University, Wuxi, Jiangsu, China

† Xingguang Lin and Dongrui Deng contributed equally to this work and shared correspondence

* Correspondence: [tjdengdongrui@126.com](mailto:tjdengdongrui@126.com)

Figure 1: Causal directed acyclic graph (DAG)


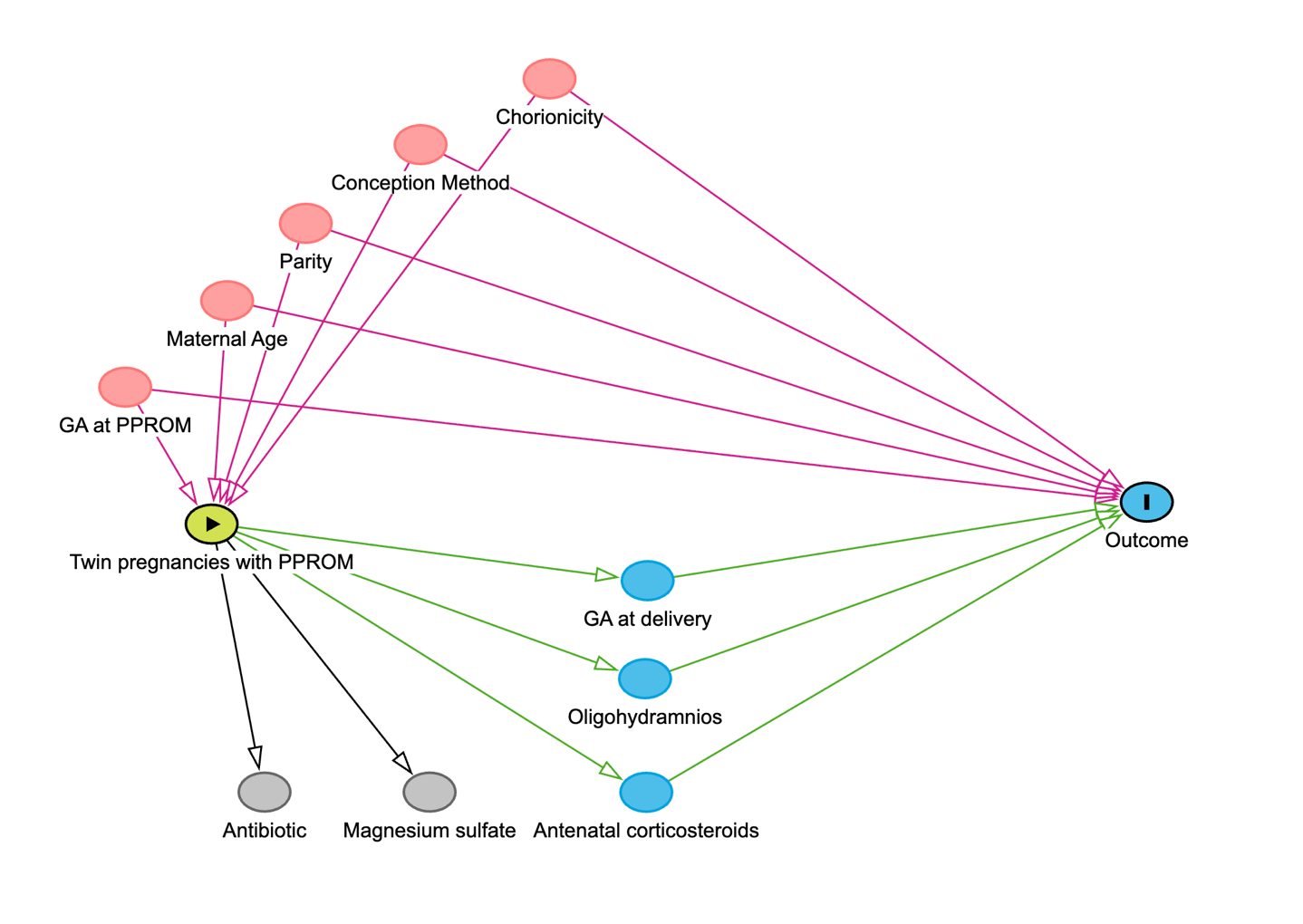


Table S1: Definitions of Neonatal Outcomes

| **Variables** | **Definition** |
| --- | --- |
| Respiratory Distress Syndrome (RDS) | A condition caused by pulmonary immaturity and surfactant deficiency, leading to respiratory insufficiency soon after birth. Diagnosis based on the following 3 aspects:   1. Clinical symptoms: worsening clinical signs such as tachypnea (respiratory rate > 60 breaths per minute), retractions, grunting, and cyanosis with a requirement of supplement oxygen; 2. Arterial blood gas analysis: typically reveals hypoxemia, hypercapnia, and acidosis; 3. Chest X-ray data: shows classic patterns like granular densities, air bronchograms, or, in severe cases, a complete "white-lung" appearance. |
| Bronchopulmonary Dysplasia (BPD) | Diagnosed per the NICHD (2001) criteria for infants born at <32 weeks' gestation: requirement of supplemental oxygen for at least 28 days. Severity is graded at 36 weeks' postmenstrual age: Mild (breathing room air), Moderate (requiring FiO₂ < 0.30), Severe (requiring FiO₂ ≥ 0.30 or positive pressure/mechanical ventilation). |
| Severe Intraventricular Hemorrhage  (IVH 3-4) | Grade III or IV IVH, defined according to Papile's classification, diagnosed by ultrasound. |
| Necrotizing Enterocolitis (NEC) | characterized by mucosal or transmural necrosis of the intestine, with clinical signs including feeding intolerance, abdominal distension, and bloody stools, often confirmed radiographically by pneumatosis intestinalis, diagnosed as Bell's stage II or greater. |
| Retinopathy Of Prematurity (ROP) | characterized by abnormal blood vessel growth in the retina.  The condition is driven by interrupted normal retinal vascular development after premature birth, followed by pathological neovascularization, which can lead to tractional retinal detachment and blindness. |
| Early-Onset Sepsis (EOS) | Diagnosed from positive bacteriology findings in blood or cerebrospinal fluid (laboratory confirmation) within the first 72 hours of life. |
| Late-Onset Sepsis (LOS) | Diagnosed from positive bacteriology findings in blood or cerebrospinal fluid (laboratory confirmation) occurring after 72 hours of life. |
| Patent Ductus Arteriosus (PDA) | Hemodynamically significant PDA (hsPDA) is diagnosed based on clinical signs (e.g., increased respiratory support, metabolic acidosis, tachycardia, wide pulse pressure >25 mmHg, cardiac murmur) and confirmed by echocardiographic criteria: ductal diameter > 1.5 mm, left-to-right shunt, and left atrium-to-aortic root ratio > 1.4. |
| Neonatal Pneumonia | A lower respiratory tract infection characterized by clinical signs such as tachypnea (respiratory rate > 60 breaths/min), cough, grunting, retractions, and abnormal auscultatory findings (rales/rhonchi), supported by radiological evidence of pulmonary infiltrates, consolidation, or interstitial changes. |
| Neonatal Hypoglycemia | Blood glucose level (BGL) at any time <2.2mmol/L (40mg/dl). |
| Neonatal Hyperbilirubinemia | Elevated levels of total serum bilirubin greater than the 95th percentile for the corresponding hour-specific age. |
| Neonatal Anaemia | Diagnosis is based on laboratory measurement of hemoglobin or hematocrit, with transfusion thresholds defined for specific patient populations, such as a threshold of Hb < 70 g/L for stable pediatric patients without active bleeding. |
| Neonatal Death | The death of a live-born infant within the first 28 days of life. |
| NICU admission | Common indications include: gestational age <34 weeks, birth weight <2000g, need for respiratory support, major surgery, severe asphyxia, seizures, or other critical conditions requiring intensive care. |
