# Supplementary material for: Maintenance tocolysis in twin pregnancies after preterm premature rupture of membranes and neonatal outcomes: a retrospective cohort study
Source: BMC Pregnancy Childbirth. 2026 Apr 29;26:647. doi: 10.1186/s12884-026-09144-8 (PMC13267740; doi:10.1186/s12884-026-09144-8)
Supplement: Supplementary file 2 — Supplementary Material 2. [file 12884_2026_9144_MOESM2_ESM.docx]

**Maintenance tocolysis in twin pregnancies after preterm premature rupture of membranes and neonatal outcomes: a retrospective cohort study**

Leakana Praseth^a^, Dan Lv ^a,b^, Shiyao Chen^a^, Xufang Li^a^, Jiaqi Han^a^, Xinyu He^a^, Xingguang Lin^a,^ ^†^, Dongrui Deng ^a,^ ^†, *^

**Table S1:** Association Between Maintenance Tocolysis and Outcomes, Stratified by GA at PPROM

| **Outcome** | **Early PPROM (n=81)** | |  | **Late PPROM (n=30)** | |
| --- | --- | --- | --- | --- | --- |
|  | **Maintenance Tocolysis vs.**  **Non-maintenance Tocolysis (ref)** | |  | **Maintenance Tocolysis vs.**  **Non-maintenance Tocolysis (ref)** | |
|  | Adjusted β (95% CI) | *P*-value |  | Adjusted β (95% CI) | *P*-value |
| GA at delivery ^a^ | 0.85 (0.16,1.54) | **0.016** |  | 0.77 (0.35,1.20) | **<.001** |
| Latency prolongation ^a^ | 6.01 (1.18,10.85) | **0.015** |  | 5.34 (2.44,8.25) | **<.001** |
| Birthweight ^b^ | 10.78 (-55.20,76.77) | 0.749 |  | -74.63 (-183.33,34.07) | 0.178 |
| Birthweight percentile ^c^ | 0.40 (-8.53,9.33) | 0.930 |  | -17.04 (-30.27,-3.82) | **0.012** |
| Birthweight Z-score ^c^ | -0.01 (-0.42,0.40) | 0.964 |  | -0.38 (-0.88,0.12) | 0.138 |
| Neonatal hospital stay ^a^ | -0.08 (-0.35,0.20) | 0.582 |  | 0.10 (-0.25,0.46) | 0.571 |
| **Neonatal complications ^a^** | aRR/aOR (95% CI) | *P*-value |  | aRR/aOR (95% CI) | *P*-value |
| NICU admission | 0.97 (0.95,1.00) | 0.092 |  | 0.73 (0.49,1.08) | 0.117 |
| Hyperbilirubinemia | 1.28 (0.48,3.37) | 0.621 |  | 0.21 (0.05,0.98) | **0.047** |
| Pneumonia | 1.07 (0.47,2.45) | 0.864 |  | 0.66 (0.01,53.72) | 0.852 |
| RDS | 0.88 (0.33,2.36) | 0.803 |  | 0.17 (0.02,1.67) | 0.130 |
| Anaemia | 0.66 (0.29,1.53) | 0.336 |  | 0.21 (0.02,2.09) | 0.185 |
| PDA | 0.24 (0.07,0.81) | **0.021** |  | NA | NA |
| BPD | 0.60 (0.22,1.61) | 0.312 |  | NA | NA |
| Hypoglycemia | 5.23 (0.55,49.66) | 0.149 |  | 1.52 (0.24,9.72) | 0.657 |
| IVH (3-4) | 0.43 (0.12,1.55) | 0.196 |  | 0.07 (0.005,0.90) | **0.042** |
| ROP | 0.02 (0.00,1.80) | 0.086 |  | NA | NA |
| NEC | 0.36 (0.06,2.11) | 0.259 |  | NA | NA |
| Early-onset Sepsis | 0.57 (0.08,4.28) | 0.588 |  | NA | NA |
| Late-onset Sepsis | 0.30 (0.05,1.65) | 0.167 |  | NA | NA |
| Neonatal death | 0.33 (0.08,1.30) | 0.113 |  | NA | NA |

CI, confidence interval; aRR, adjusted relative risk; aOR, adjusted odds ratio; ref, reference group. The neonatal outcome was considered present if it occurred in at least one neonate of the twin pair.

Subgroups by GA at PPROM: Early PPROM subgroup (24 0/7 to 31 6/7 weeks) n=81 pregnancies (Non-maintenance: n=28; Maintenance: n=53); 144 neonates (49 in Non-maintenance; 95 in Maintenance tocolysis). Late PPROM subgroup (32 0/7 to 33 6/7 weeks) n=30 pregnancies (Non-maintenance: n=17; Maintenance: n=13); 54 neonates (28 in Non-maintenance; 26 in Maintenance tocolysis).

^a^ Adjusted for maternal characteristics (age, method of conception, parity), chorionicity, GA at PPROM, and tocolytic therapy.

^b^ Adjusted for maternal characteristics (age, method of conception, parity), chorionicity, GA at delivery, tocolytic therapy, and fetal sex.

^c^ Adjusted for maternal characteristics (age, method of conception, parity), chorionicity, tocolytic therapy, and fetal sex.

**Table S2**. Outcomes by Tocolysis Duration in Twin Pregnancies with PPROM Undelivered at 48 Hours

| **Outcome** | **Short-term tocolysis vs.**  **No tocolysis (ref)** | |  | **Long-term tocolysis vs.**  **No tocolysis (ref)** | |  | **Long-term tocolysis vs.**  **Short-term tocolysis (ref)** | |
| --- | --- | --- | --- | --- | --- | --- | --- | --- |
|  | Adjusted β (95% CI) | *P* |  | Adjusted β (95% CI) | *P* |  | Adjusted β (95% CI) | *P* |
| GA at delivery ^a^ | -0.18 (-1.02,0.67) | 0.683 |  | 0.62 (-0.16,1.40) | 0.117 |  | 0.80 (0.22, 1.37) | **0.007** |
| Latency prolongation ^a^ | -1.29 (-7.19,4.61) | 0.668 |  | 4.32 (-1.12,9.76) | 0.119 |  | 5.61 (1.59,9.64) | **0.006** |
| Birthweight ^b^ | -17.41 (-113.48,78.66) | 0.772 |  | -23.45 (-104.35,57.46) | 0.570 |  | -6.04 (-79.97,67.90) | 0.873 |
| Birthweight percentile ^c^ | 4.12 (-7.74,15.98) | 0.496 |  | 0.58 (-9.75,10.91) | 0.913 |  | -3.54 (-12.64,5.55) | 0.445 |
| Birthweight Z-score ^c^ | -0.11 (-0.58,0.37) | 0.659 |  | -0.14 (-0.54,0.26) | 0.506 |  | -0.03 (-0.44,0.38) | 0.889 |
| Neonatal hospital stay ^a^ | 0.09 (-0.20,0.37) | 0.545 |  | 0.00 (-0.25,0.25) | 0.978 |  | -0.09 (-0.35,0.16) | 0.479 |
| **Neonatal complications ^a^** | aRR/aOR (95% CI) | *P* |  | aRR/aOR (95% CI) | *P* |  | aRR/aOR (95% CI) | *P* |
| NICU admission | 1.04 (0.90,1.20) | 0.635 |  | 0.95 (0.80,1.12) | 0.539 |  | 0.91 (0.85,0.99) | **0.019** |
| Hyperbilirubinemia | 0.49 (0.13,1.79) | 0.279 |  | 0.54 (0.17,1.71) | 0.294 |  | 1.10 (0.45,2.69) | 0.835 |
| Pneumonia | 1.02 (0.30,3.45) | 0.976 |  | 1.08 (0.33,3.56) | 0.902 |  | 1.06 (0.48,2.32) | 0.888 |
| RDS | 0.57 (0.14,2.31) | 0.433 |  | 0.48 (0.13,1.69) | 0.250 |  | 0.83 (0.32,2.20) | 0.714 |
| Anaemia | 2.70 (0.67,10.85) | 0.162 |  | 1.38 (0.34,5.59) | 0.650 |  | 0.51 (0.23,1.13) | 0.098 |
| PDA | 1.03 (0.25,4.25) | 0.971 |  | 0.21 (0.04,1.07) | 0.060 |  | 0.20 (0.06,0.70) | **0.012** |
| BPD | 2.57 (0.73,9.03) | 0.142 |  | 1.36 (0.44,4.15) | 0.593 |  | 0.53 (0.17,1.61) | 0.263 |
| Hypoglycemia | 0.93 (0.06,14.19) | 0.959 |  | 3.56 (0.44,28.84) | 0.233 |  | 3.83 (0.77,19.01) | 0.100 |
| IVH (3-4) | 1.43 (0.24,8.65) | 0.694 |  | 0.61 (0.12,3.11) | 0.552 |  | 0.43 (0.12,1.52) | 0.188 |
| ROP | 0.16 (0.01,3.31) | 0.236 |  | 0.003 (0.000,0.46) | **0.023** |  | 0.02 (0.00,5.77) | 0.179 |
| NEC | NA | NA |  | NA | NA |  | NA | NA |
| Early-onset Sepsis | NA | NA |  | NA | NA |  | NA | NA |
| Late-onset Sepsis | 0.66 (0.06,7.07) | 0.735 |  | 0.25 (0.02,2.87) | 0.264 |  | 0.37 (0.05,2.57) | 0.315 |
| Neonatal death | 2.40 (0.30,19.26) | 0.410 |  | 0.65 (0.08,5.49) | 0.689 |  | 0.27 (0.06,1.22) | 0.089 |

CI, confidence interval; aRR, adjusted relative risk; aOR, adjusted odds ratio; ref, reference group. The neonatal outcome was considered present if it occurred in at least one neonate of the twin pair.

Stratified by tocolysis based on duration: No tocolysis (n=15 pregnancies, 24 neonates); Short-term tocolysis (n=30 pregnancies, 53 neonates); Long-term tocolysis (n=66 pregnancies, 121 neonates).

^a^ Adjusted for maternal characteristics (age, method of conception, parity), chorionicity, GA at PPROM, and tocolytic therapy.

^b^ Adjusted for maternal characteristics (age, method of conception, parity), chorionicity, GA at delivery, tocolytic therapy, and fetal sex.

^c^ Adjusted for maternal characteristics (age, method of conception, parity), chorionicity, tocolytic therapy, and fetal sex.

**Table S3**. Association Between Maintenance Tocolysis and Outcomes, According to Tocolytic Agents

| **Outcome** | **Tocolytic Group 1 (n=65)** | |  | **Tocolytic Group 2 (n=30)** | |
| --- | --- | --- | --- | --- | --- |
|  | **Maintenance Tocolysis vs.**  **Non-maintenance Tocolysis (ref)** | |  | **Maintenance Tocolysis vs.**  **Non-maintenance Tocolysis (ref)** | |
|  | Adjusted β (95% CI) | *P*-value |  | Adjusted β (95% CI) | *P*-value |
| GA at delivery ^a^ | 0.76 (-0.02,1.53) | 0.057 |  | 0.97 (-0.45,2.39) | 0.182 |
| Latency prolongation ^a^ | 5.30 (-0.14,10.75) | 0.056 |  | 6.80 (-3.16,16.75) | 0.181 |
| Birthweight ^b^ | -46.59 (-148.79,55.60) | 0.372 |  | 12.62 (-132.03,157.26) | 0.864 |
| Birthweight percentile ^c^ | -8.17 (-20.32,3.97) | 0.187 |  | 1.73 (-17.93,21.40) | 0.863 |
| Birthweight Z-score ^c^ | -0.21 (-0.77,0.34) | 0.453 |  | 0.06 (-0.81,0.94) | 0.885 |
| Neonatal hospital stay ^a^ | 0.02 (-0.26,0.30) | 0.910 |  | -0.03 (-0.65,0.58) | 0.918 |
| **Latency period ^a^** | aOR (95% CI) | *P*-value |  |  |  |
| Up to 72h | reference |  |  |  |  |
| 3 - 7days | 3.95 (0.63,24.76) | 0.142 |  |  |  |
| Over 7days | 16.88 (2.36,120.63) | **0.005** |  |  |  |
|  |  |  |  |  |  |
| 3 - 7days | reference |  |  |  |  |
| Over 7days | 4.27 (0.90,20.27) | 0.068 |  |  |  |
| **Neonatal complications ^a^** | aRR/aOR (95% CI) | *P*-value |  | aRR/aOR (95% CI) | *P*-value |
| NICU admission | 0.88 (0.79,0.99) | **0.030** |  | 0.96 (0.89,1.04) | 0.310 |
| Hyperbilirubinemia | 1.18 (0.41,3.43) | 0.762 |  | 3.17 (0.38,26.74) | 0.289 |
| Pneumonia | 1.24 (0.46,3.31) | 0.669 |  | 0.34 (0.03,3.54) | 0.366 |
| RDS | 0.72 (0.20,2.56) | 0.618 |  | 1.14 (0.18,7.35) | 0.889 |
| Anaemia | 0.77 (0.26,2.30) | 0.635 |  | 0.52 (0.09,3.08) | 0.473 |
| PDA | 0.27 (0.06,1.22) | 0.089 |  | 0.08 (0.00,7.24) | 0.268 |
| BPD | 0.49 (0.11,2.23) | 0.358 |  | 0.82 (0.06,10.53) | 0.876 |
| Hypoglycemia | 2.17 (0.46,10.18) | 0.324 |  | NA | NA |
| IVH (3-4) | 0.68 (0.12,3.89) | 0.664 |  | 0.003 (0.000,0.58) | **0.031** |
| ROP | NA | NA |  | 0.74 (0.09,5.85) | 0.772 |
| NEC | NA | NA |  | 0.33 (0.05,2.30) | 0.260 |
| Early-onset Sepsis | NA | NA |  | 0.13 (0.01,1.75) | 0.124 |
| Late-onset Sepsis | NA | NA |  | 0.45 (0.04,5.24) | 0.526 |
| Neonatal death | 0.86 (0.06,11.53) | 0.909 |  | 0.01 (0.001,0.18) | **0.002** |

CI, confidence interval; aRR, adjusted relative risk; aOR, adjusted odds ratio; ref, reference group. The neonatal outcome was considered present if it occurred in at least one neonate of the twin pair.

Tocolytic Agents: Group 1 (beta-adrenergic receptor agonist, ritodrine): 65 pregnancies (Non-maintenance: n=19; Maintenance: n=46); 120 neonates (Non-maintenance: n=33; Maintenance: n=87); Group 2 (calcium-channel blockers [CCB], nifedipine/oxytocin receptor antagonist, atosiban): 30 pregnancies (Non-maintenance: n=10; Maintenance: n=20); 52 neonates (Non-maintenance: n=18; Maintenance: n=34).

^a^ Adjusted for maternal characteristics (age, method of conception, parity), chorionicity, GA at PPROM, and tocolytic therapy.

^b^ Adjusted for maternal characteristics (age, method of conception, parity), chorionicity, GA at delivery, tocolytic therapy, and fetal sex.

^c^ Adjusted for maternal characteristics (age, method of conception, parity), chorionicity, tocolytic therapy, and fetal sex.

**Table S4.** Comparative effectiveness of the Two Maintenance Tocolytic agents

| **Outcome** | **Maintenance Tocolytic Group 2 (n=20) vs.**  **Maintenance Tocolytic Group 1 (n=46, ref)** | |
| --- | --- | --- |
|  | Adjusted β (95% CI) | *P*-value |
| GA at delivery ^a^ | -0.62 (-1.57,0.32) | 0.198 |
| Latency prolongation ^a^ | -4.36 (-10.96,2.25) | 0.196 |
| Birthweight ^b^ | 20.33 (-103.72,144.38) | 0.748 |
| Birthweight percentile ^c^ | 1.16 (-12.20,14.52) | 0.865 |
| Birthweight Z-score ^c^ | 0.05 (-0.54,0.65) | 0.862 |
| Neonatal hospital stay ^a^ | 0.16 (-0.15,0.47) | 0.308 |
| **Neonatal complications ^a^** | aRR/aOR (95% CI) | *P*-value |
| NICU admission | 1.07 (0.95,1.20) | 0.252 |
| Hyperbilirubinemia | 1.06 (0.35,3.25) | 0.917 |
| Pneumonia | 0.10 (0.02,0.51) | **0.006** |
| RDS | 3.21 (0.74,13.80) | 0.118 |
| Anaemia | 1.76 (0.43,7.17) | 0.427 |
| PDA | 2.26 (0.47,10.93) | 0.310 |
| BPD | 1.11 (0.22,5.72) | 0.897 |
| Hypoglycemia | 5.18 (0.74,35.98) | 0.097 |
| IVH (3-4) | 0.20 (0.02,1.79) | 0.150 |
| ROP | NA | NA |
| NEC | 1.23 (0.20,7.60) | 0.820 |
| Early-onset Sepsis | 1.03 (0.12,9.11) | 0.976 |
| Late-onset Sepsis | 24.75 (5.09,120.48) | **<.001** |
| Neonatal death | 1.44 (0.35,5.94) | 0.613 |

CI, confidence interval; aRR, adjusted relative risk; aOR, adjusted odds ratio; ref, reference group. The neonatal outcome was considered present if it occurred in at least one neonate of the twin pair.

Maintenance Tocolytic Group 1 (beta-adrenergic receptor agonists, ritodrine), 46 pregnancies (87 neonates); Maintenance Tocolytic Group 2 (calcium-channel blockers [CCB], nifedipine + oxytocin receptor antagonist, atosiban), 20 pregnancies (34 neonates).

^a^ Adjusted for maternal characteristics (age, method of conception, parity), chorionicity, GA at PPROM, and tocolytic therapy.

^b^ Adjusted for maternal characteristics (age, method of conception, parity), chorionicity, GA at delivery, tocolytic therapy, and fetal sex.

^c^ Adjusted for maternal characteristics (age, method of conception, parity), chorionicity, tocolytic therapy, and fetal sex.
